# Enhanced 2,3-butanediol production from biodiesel-derived glycerol by engineering of cofactor regeneration and manipulating carbon flux in *Bacillus amyloliquefaciens*

**DOI:** 10.1186/s12934-015-0317-2

**Published:** 2015-08-22

**Authors:** Taowei Yang, Zhiming Rao, Xian Zhang, Meijuan Xu, Zhenghong Xu, Shang-Tian Yang

**Affiliations:** The Key Laboratory of Industrial Biotechnology, Ministry of Education, School of Biotechnology, Jiangnan University, Wuxi, Jiangsu Province 214122 China; Laboratory of Pharmaceutical Engineering, School of Pharmaceutical Science, Jiangnan University, Wuxi, Jiangsu Province 214122 China; Department of Chemical and Biomolecular Engineering, The Ohio State University, Columbus, OH 43210 USA; School of Biotechnology, Jiangnan University, 1800 Lihu Avenue, Wuxi, Jiangsu 214122 People’s Republic of China

**Keywords:** 2,3-butanediol, Glycerol, Cofactor regeneration, Manipulating carbon flux, *Bacillus amyloliquefaciens*

## Abstract

**Background:**

*Bacillus amyloliquefaciens* B10-127 exhibited an excellent ability for industrial-scale microbial fermentation of 2,3-butanediol (2,3-BD) from biodiesel-derived glycerol. However, the accumulation of by-products (acetoin, acetoin, lactate and succinate) and the 2,3-BD yield remains prohibitively low for commercial production.

**Results:**

Several strategies were developed to manipulate the carbon flux to 2,3-BD branch in a designed *B. amyloliquefaciens*. Firstly, extra copies of NADH/NAD^+^ regeneration system were introduced into *B. amyloliquefaciens* by co-overproduction of glycerol dehydrogenase and acetoin reductase, which resulting in improvement of 2,3-BD production and suppression of by-products accumulation. Subsequently, the transcriptional regulator ALsR under the control of a moderate promoter P_bdhA_ was introduced into *B. amyloliquefaciens*, which increased carbon flux to 2,3-BD branch. Finally, a three-stage dissolved oxygen control strategy were proposed based on analysis of the characteristic of 2,3-BD fermentation, and a two-stage pH control strategy were proposed based on different pH preferences of ACR for reduction and oxidation. Following these strategies, a high titer (102.3 g/L), yield (0.44 g/g), and productivity (1.16 g/L/h) of 2,3-BD were achieved.

**Conclusions:**

To our knowledge, this is the highest reported 2,3-BD production using biodiesel-derived glycerol as substrate, and this designed *B. amyloliquefaciens* should be an excellent candidate for producing 2,3-BD on an industrial scale.

## Background

2,3-Butanediol (2,3-BD) has potential applications in the manufacture of foods, pharmaceuticals fumigants, printing inks, moistening and softening agents, plasticizers [1]. Interest in microbial production of 2,3-BD has increased significantly because 2,3-BD has a wide range of industrial applications, and microbial production will alleviate the dependence on oil supply for the production of platform chemicals [2, 3].

To date, many studies on 2,3-BD production have focused on sugar fermentation. Despite the high-effective productivities that have been achieved via the conversion of glucose [4], the relatively high cost of conventional sugar substrates is still viewed as a major factor during 2,3-BD fermentation. Therefore, 2,3-BD production using cheaper alternative biomass-derived substrates under proper conditions is of high priority [2]. Biodiesel production from plant oils and animal fats reportedly generate large quantities of by-product waste glycerol [5]. For example, approximately 10 % of the biodiesel weight produced is waste glycerol [6]. In some European countries, the production of glycerol has increased significantly due to biodiesel uptake. However, those biodiesel companies have severe problems getting rid of excess glycerol and disposal is quite expensive. The collapse of glycerol prices causes major problems to these companies [7]. Since glycerol can be used as a carbon source in industrial microbiology, this by-product adds value to the productive chain of the biodiesel industry, contributing to their competitiveness [6]. Raw non-purified glycerol is an economical substitute for pure glycerol as a fermentation substrate. Therefore, converting the vast amounts of glycerol into cost-effective commercial products is an industrial priority. Some researchers have reported that *Klebsiella* strains can catalyze pure glycerol into 2,3-BD, but these reactions generate large quantities of 1,3-PD [8, 9]. Importantly, because *Klebsiella pneumoniae* is a pathogenic microorganism, it does not conform to the safety regulations of industrial-scale fermentation [3]. Metsoviti et al. [10] obtained a 2,3-BD concentration of 22 g/l and with a relatively high conversion yield on glycerol consumed of 0.40 g/g with a newly isolated *Enterobacter aerogenes* FMCC-10; however, the efficiency of the production was still much too low for an economic process.

Previously, we reported that *B. amyloliquefaciens* readily produces 2,3-BD from biodiesel-derived glycerol in the presence of beet molasses as a co-substrate [11]. In fed-batch fermentation, 2,3-BD production (83.3 g/L) from waste glycerol reached the highest level reported to date, but fermentation was accompanied by undesirably large production of acetoin, lactate, acetate, and succinate. However, both raw glycerol and molasses are easily available by-products from plant (mostly) biomass conversion, and they represent abundant renewable feed stocks and furthermore, they need no pretreatment before fermentation processes. So microbial production of 2,3-BD from these raw materials is potentially economically feasible if bacterial strain used for this purpose produces at least 100 g 2,3-BD per L (the recovery of 2,3-BD from culture broth is troublesome and usually energy-consuming distillation is necessary). However, neither process conditions have been optimized (it was just comparison of the effect of pure glucose or sucrose, or molasses on 2,3-BD biosynthesis yield) nor the strain was improved by genetic engineering methods.

**Scheme 1:**
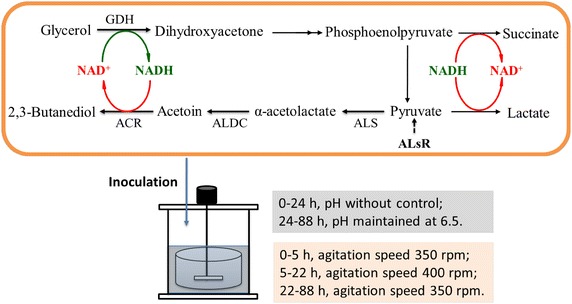
The strategy for designed *B. amyloliquefaciens* to manipulate the carbon flux to 2,3-BD branch. To make the 2,3-BD branch gains a competitive advantage over the end products of pyruvate-deriving pathways (such as acetic acid, lactic acid and succinic acid), several strategies were developed to manipulate the carbon flux to 2,3-BD branch in a designed *B. amyloliquefaciens*. Firstly, extra copies of NADH/NAD^+^ regeneration system were introduced into *B. amyloliquefaciens* by co-overproduction of glycerol dehydrogenase and acetoin reductase. Subsequently, the transcriptional regulator ALsR under the control of a moderate promoter P_bdhA_ was introduced into *B. amyloliquefaciens*. Finally, a three-stage dissolved oxygen control strategy were proposed based on analysis of the characteristic of 2,3-BD fermentation, and a two-stage pH control strategy were proposed based on different pH preferences of ACR for reduction and oxidation

The 2,3-BD pathway has been studied in various bacteria [12]. As shown in Scheme 1, assimilation of glycerol to produce 2,3-BD is also an oxido-reduction-associated process. In the oxidative pathway, the NAD^+^-dependent glycerol dehydrogenase (GDH) oxidizes glycerol to dihydroxyacetone, which is subsequently oxidized to pyruvate. Acetolactate synthase (ALS) catalyzes the in vivo coupling of two pyruvate molecules to form acetolactate, which is then decarboxylated to acetoin by acetolactate decarboxylase (ALDC). Finally, acetoin is reduced to 2,3-BD by an NADH-dependent acetoin reductase (ACR) [13]. Bacterial strains may accumulate acetoin for several reasons. One factor that limits acetoin degradation is low levels of ACR, assumed as the rate-limiting factor in the conversion of acetoin into 2,3-BD. Alternatively, low levels of NADH may limit the ACR reaction, since this coenzyme is preferentially used in 2,3-BD synthesis. However, some other by-products are also produced by *B. amyloliquefaciens*, such as succinate, lactate, and acetate, which negatively regulate the 2,3-BD yield and increase the costs of downstream separation and purification. Furthermore, large-scale microbial 2,3-BD production requires efficient and economical fermentation processes. Thus, in this work, we focused on improving strains to produce 2,3-BD with high yield, using low price substrates (raw glycerol and molasses) to lower the cost of feedstock, and optimizing the operation mode to make the process more efficient (Scheme 1).

## Results and discussion

### Over-production of glycerol dehydrogenase and its effects on 2,3-BD production

Glycerol dehydrogenase (GDH) is an important polyol dehydrogenase for glycerol metabolism in diverse microorganisms, and for value-added utilization of glycerol in the industry that catalyzes the dehydrogenation of glycerol to dihydroxyacetone. This reaction is coupled to the reduction of oxidized NAD^+^ to NADH. So, over-production of glycerol dehydrogenase could increase not only the dehydrogenation of glycerol but also the level of available NADH.

In the genome sequence of *K. pneumoniae* ATCC 25955, there are two GDHs (DhaD and GldA) [14]. So, the two enzymes were separately introduced into *B. amyloliquefaciens*, and generated recombinant strains DH and GL, respectively. As shown in Table 1, the specific activities of GDH in strains DH and GL were separately 4.76 and 3.02 folds higher than in the parental strain. Also, the highest 2,3-BD concentration were increased by 10.7 % with strain DH and 6.35 % with strain GL, which suggested that overproduction of DhaD was more efficient for 2,3-BD production. Wang et al. [14] found that DhaD is highly induced by glycerol, and apart from catalyzing the dehydrogenation of glycerol to dihydroxyacetone, it also could catalyze the reduction of acetoin to 2,3-BD in the presence of NADH. In other words, DhaD plays a dual role in glycerol metabolism and 2,3-butanediol formation. Therefore, DhaD over-eproduction might enhance 2,3-BD production by increasing not only the level of available NADH but also catalytic activity of 2,3-BD formation.

### Introduction of extra copies of DhaD/ACR enzymes into *B. amyloliquefaciens* and their effects on 2,3-BD production

The NAD^+^-dependent DhaD oxidizes glycerol to dihydroxyacetone, with concomitant reduction of NAD^+^ to NADH. In contrast, the NADH-dependent ACR reduces acetoin to 2,3-BD, with concomitant oxidation of NADH to NAD^+^. So, co-overproduction of DhaD and ACR may enhance 2,3-BD production [15]. Inspired by this idea, the *dhaD* gene harbored in pMA5-*acr* was overexpressed in the strain GA. The plasmid genetic rate remained about 95 %, indicating that the pMA5-*acr*-HapII-*dhaD* was stably expressed in the strain GA. The specific activities of GDH (0.67 ± 0.06 U/mg) and ACR (0.58 ± 0.05 U/mg) in the strain GA were 4.58-fold and 3.66-fold higher than in the strain B10-127, respectively.

**Fig. 1:**
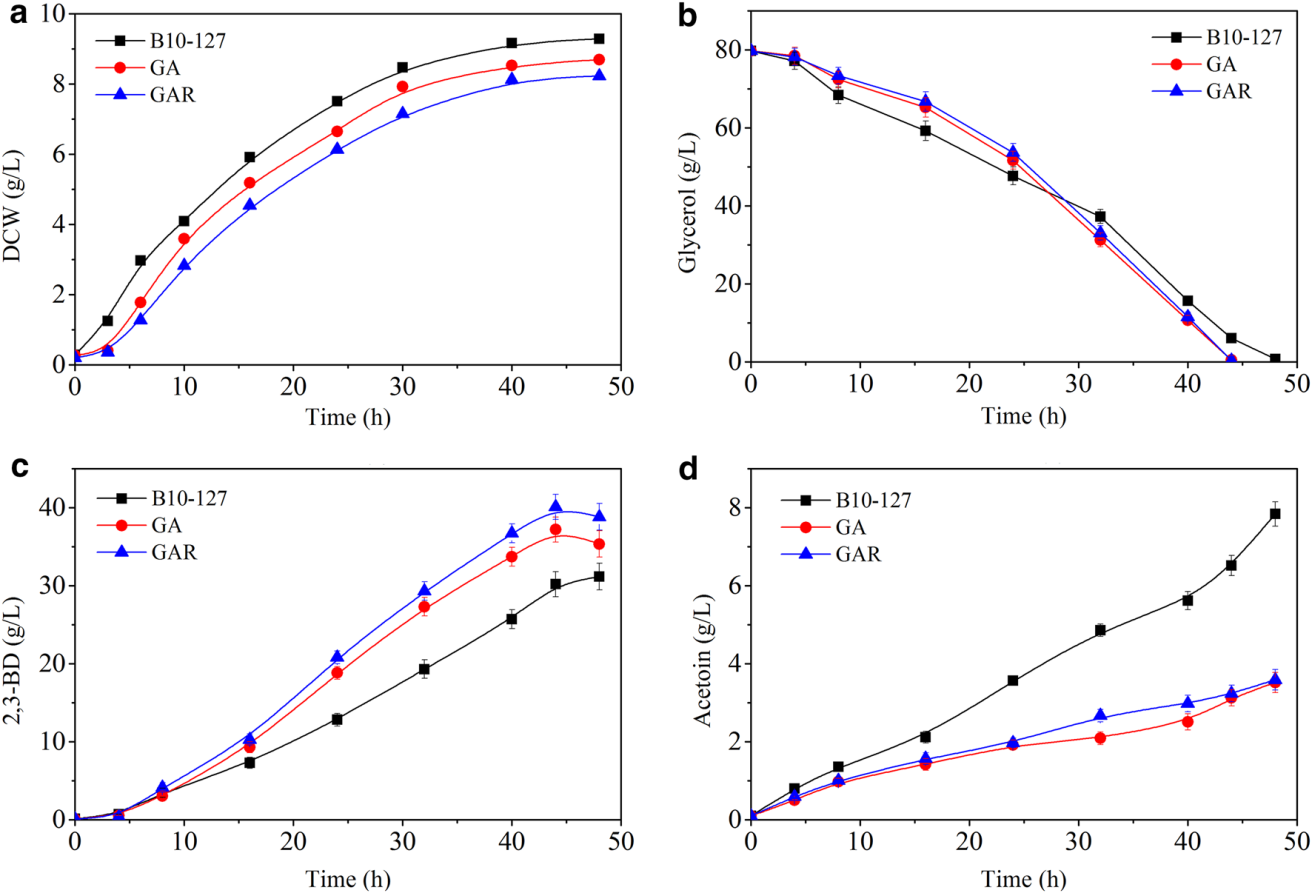
Time profiles of 2,3-BD fermentation (**a** cell growth; **b** glycerol consumption; **c** 2,3-BD production; **d** acetoin formation). Batch fermentation was carried out at 37 °C in a 5-L bioreactor containing 2.5-L initial medium [95 g/L co-substrate; molasses to crude glycerol ratio 1.5:8 (w/w)] at 37 °C, agitation speed 350 rpm, and airflow rate 0.66 vvm

The effects co-overexpression of *dhaD* and *acr* on cell growth and 2,3-BD production were also investigated. As shown in Fig. 1a, the strain GA grew at a slightly lower rate compared to that of the parent strain (B10-127), suggesting that cell growth was slightly inhibited by the over-production of DhaD/ACR. As shown in Fig. 1b, the strain GA consumes glycerol more slowly than the parental strain during the exponential growth phase. However, the fermentation rate of the engineered strain GA remarkably increased in the stationary phase, with consequent reduction in fermentation time. Furthermore, co-overproduction of DhaD and ACR in *B. amyloliquefaciens* increased the highest 2,3-BD titer by 13.6 %, while decreasing the acetoin concentration by 64.6 % (see Fig. 1c, d). In addition, other by-products production, such as acetate, lactate and succinate, were also suppressed. However, in both parental and engineered strains, the intracellular NADH and NAD^+^ concentration had no difference during the glycerol fermentation (data not shown), possibly because introducing extra copies of DhaD/ACR enzymes into *B. amyloliquefaciens* accelerated the NADH/NAD^+^ regeneration rate without influencing the NAD^+^/NADH levels. Two reasons may account for this phenomenon. First, over-production of NAD^+^-dependent DhaD increased the rate of glycerol consumption and NADH level. Second, in the glycerol metabolism, the 2,3-BD branch is primarily responsible for oxidizing NADH, and when NADH-dependent ACR is overproduced, the 2,3-BD branch gains a competitive advantage over the end products of pyruvate-deriving pathways (such as lactic acid and succinic acid), through the enhanced availability of NADH. So, when extra copies of DhaD/ACR enzymes were introduced into *B. amyloliquefaciens*, it was found that overexpressing the NADH/NAD^+^ regeneration system effectively improved 2,3-BD production and inhibited by-products accumulation.

### Manipulating the carbon flux from pyruvic acid to 2,3-butanediol branch by moderate expression of the transcriptional regulator ALsR

There are three key enzymes involved in 2,3-BD branch, i.e. α-acetolactate synthase (ALS), α-acetolactate decarboxylase (ALDC), and acetoin reductase (ACR). To make the 2,3-BD branch gains a competitive advantage over the end products of pyruvate-deriving pathways (such as acetic acid, lactic acid and succinic acid), in our pre-test, we attempted to overexpress ALS and ALDC to enhance acetoin production. And we succeeded in increasing the activity of these enzymes by more than 50-fold, however, 2,3-BD production was not significantly enhanced and cell growth was markedly inhibited. ALS and ALDC are encoded by the *alsSD* operon in *B. subtilis* [16]. It has been reported that the transcriptional regulator ALsR is essential for the expression of *alsSD* [17] and that the disruption of *alsR* prevents the transcription of *alsSD* [16].

The recombinant plasmids pMA5-HpaII-*alsR* and pMA5-P_bdhA_-*alsR* constructed using the strong HpaII and moderate P_bdhA_ promoters to express ALsR, respectively [18]. In the preliminary study, to improve the carbon flux to 2,3-BD branch, *alsR* under the control of two different promoters (HpaII and P_bdhA_) were cloned into *B. amyloliquefaciens* B10-127, respectively. 2,3-BD production was improved by regulating ALsR expression using either of the two promoters. However, although ALsR expression was higher under the control of the stronger promoter (HpaII) than the moderate promoter (P_bdhA_), moderate enhancement of ALsR expression was more benefic to improve 2,3-BD production. Compared with the parent strain, 2,3-BD concentration increased by 9.2 % and 15.4 % under the control of and P_bdhA_, respectively. Furthermore, the rates of cell growth and glucose consumption under the control of HpaII were lower than under the control of P_bdhA_. Zhang et al. [18] also reported that moderate enhancement of ALsR expression was more efficient for acetoin (precursor of 2,3-BD) production than strong over-expression of ALsR.

So, we selected the moderate P_bdhA_ promoter to express ALsR in *B. amyloliquefaciens* GA. The plasmid genetic rate of the resulting GAR recombinant remained stable at about 95 %, indicating that the pMA5-*dhaD*-HapII-*acr*-P_bdhA_-*als*R was stably expressed in the GAR strain. The specific activities of ALS (0.82 ± 0.07 U/mg) and ALDC (0.25 ± 0.03 U/mg) in the GAR strain were respectively 1.02-fold and 1.76-fold higher than in the GA strain.

As shown in Fig. 1a, the GA strain had a faster growth rate than the recombinant strains, suggesting that ALsR expression inhibited cell growth. All of the glycerol (80 g/L) was consumed within 44 h (Fig. 1b), and strain GAR produced approximately 38.8 g/L 2,3-BD (Fig. 1c), respectively. And main by-products production, such as acetoin, acetate, lactate and succinate, were further suppressed (Table 2). The main reason might be that the 2,3-BD branch gains a competitive advantage over the end products of pyruvate-deriving pathways. Thus, an excellent 2,3-BD producer from glycerol was redesigned through introducing extra copies of DhaD/ACR enzymes and improving catalytic activities of enzymes involved in 2,3-BD synthetic branch.

### Metabolic flux redistributions

In the aerobic glycerol metabolism of *B. amyloliquefaciens*, 2,3-BD plays a major role in oxidizing NADH. To secure NADH and C, it must compete with other end products of pyruvate-deriving pathways. Thus, this study characterized the metabolic flexibilities of *B. amyloliquefaciens* in response to over-expression of the *dhaD*, *acr* and *alsR* genes. For this purpose, the concentrations of major metabolites of both strains (B10-127, GAR) were determined (titers of 2,3-BD, acetoin, succinate, lactate and acetic acid). As shown in Table 2, compared with the parent strain, the molar yield of 2,3-BD was higher (24.1 %), and the molar yields of unwanted by-products were significantly lower in the mutant strain (64.9, 55.4, 58.3 and 36.9 % for acetoin, lactate, succinate and acetate, respectively). This observation suggests that moderate enhancement of ALsR and co-overproduction of DhaD, ACR in strain B10-127 make the 2,3-BD branch gain a competitive advantage over the end products of pyruvate-deriving pathways. And this result also indicates that glycerol fluxes are redistributed in *B. amyloliquefaciens*.

### Manipulating the carbon flux from acetoin to 2,3-butanediol by using a three-stage oxygen control strategy

Oxygen supply is one of the most important variables in the 2,3-BD fermentation [2, 3]. Jansen et al. [19] found that high oxygen supply favored cell mass formation at the expense of 2,3-BD production. Decreasing the oxygen supply would increase 2,3-BD yield, but it would decrease the overall conversion rate due to lower cell concentrations [20, 21]. Therefore, it is necessary to establish a proper oxygen supply control strategy to ensure efficient 2,3-BD production.

**Fig. 2:**
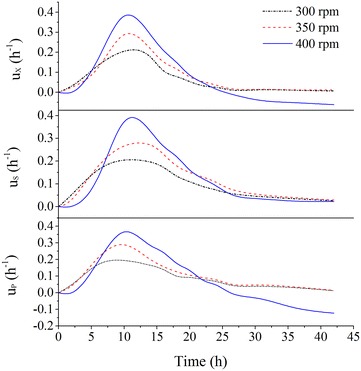
Comparison of kinetic parameters in 2,3-BD fermentation at different agitation speeds. (Batch fermentation was carried out at 37 °C in a 5-L bioreactor containing 2.5-L initial medium (95 g/L co-substrate; molasses to crude glycerol ratio 1.5:8 (w/w)) at 37 °C, and airflow rate 0.66 vvm.)

**Fig. 3:**
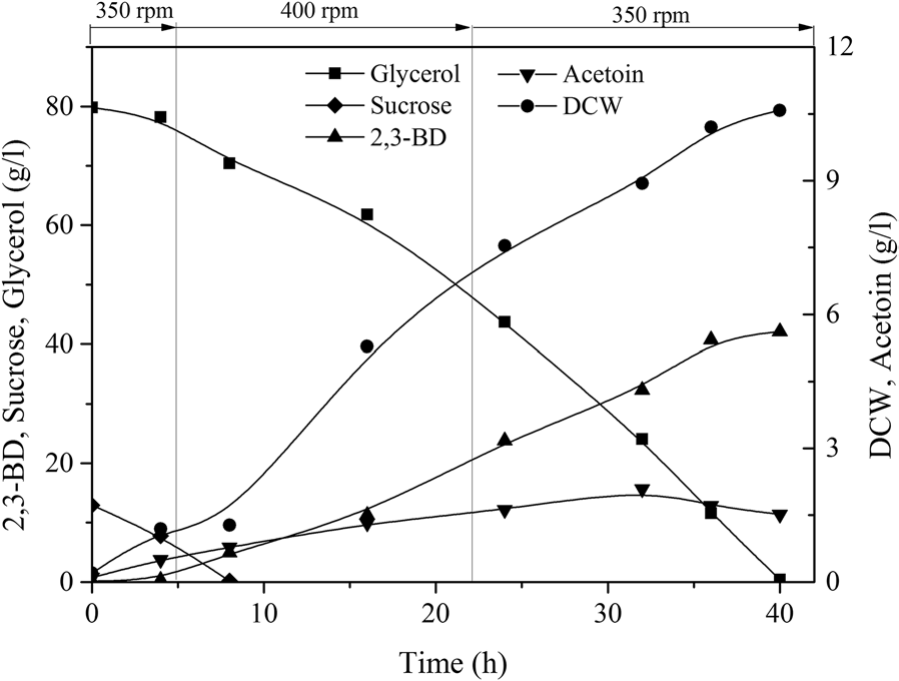
Time course of 2,3-BD fermentation by *B. amyloliquefaciens* GAR using three-stage agitation speed control strategy. (Batch fermentation was carried out at 37 °C in a 5-L bioreactor containing 2.5-L initial medium (95 g/L co-substrate; molasses to crude glycerol ratio 1.5:8 (w/w)) at 37 °C, airflow rate 0.66 vvm, and agitation speed: 0–5 h, 350 rpm; 5–22 h, 400 rpm; 22–88 h, 350 rpm.)

As it is easier to control dissolved oxygen concentration by changing agitation speed than by varying aeration rate, in this study, we investigated the characteristics of 2,3-BD fermentation by *B. amyloliquefaciens* GAR under different oxygen supply methods by changing agitation speeds. Based on the analysis of two kinetic parameters including specific cell growth rate (μ_x_) specific glycerol consumption rate (μ_s_) and specific 2,3-BD formation rate (μ_p_) (Fig. 2), a three-stage agitation speed control strategy, aimed at achieving high concentration and high yield 2,3-BD, was proposed. At the first 5 h, agitation speed was controlled at 350 rpm, subsequently agitation speed was raised to 400 rpm until 22 h, and then, agitation speed was reduced to 350 rpm (Fig. 3). Finally, the maximum concentration of 2,3-BD reached 42.6 g/L, which were 9.85 % over the best results controlled by constant agitation speeds. What’s more, titer of acetoin was reduced by 61.5 %. The proposed three-stage agitation speed control strategy was therefore proved to be successful to enhance 2,3-BD production. The idea developed in this paper could be applied to the other industrial biotechnological process to achieve high product concentration and high yield simultaneously.

Some other parameter OTR [22], k_L_a [23], OUR [24] and RQ [25, 26] guided oxygen supply control strategies were successfully applied in 2,3-BD fermentation and proved to be effective. However, the parameters of OTR, k_L_a, OUR and RQ are not easy to control, thus restricting the application of those strategies [20]. In this study, a simple oxygen supply method based on agitation speed control was set up to realize efficient 2,3-BD fermentation.

### Manipulating the carbon flux from acetoin to 2,3-butanediol by using a two-stage pH control strategy in fed-batch fermentation

**Fig. 4:**
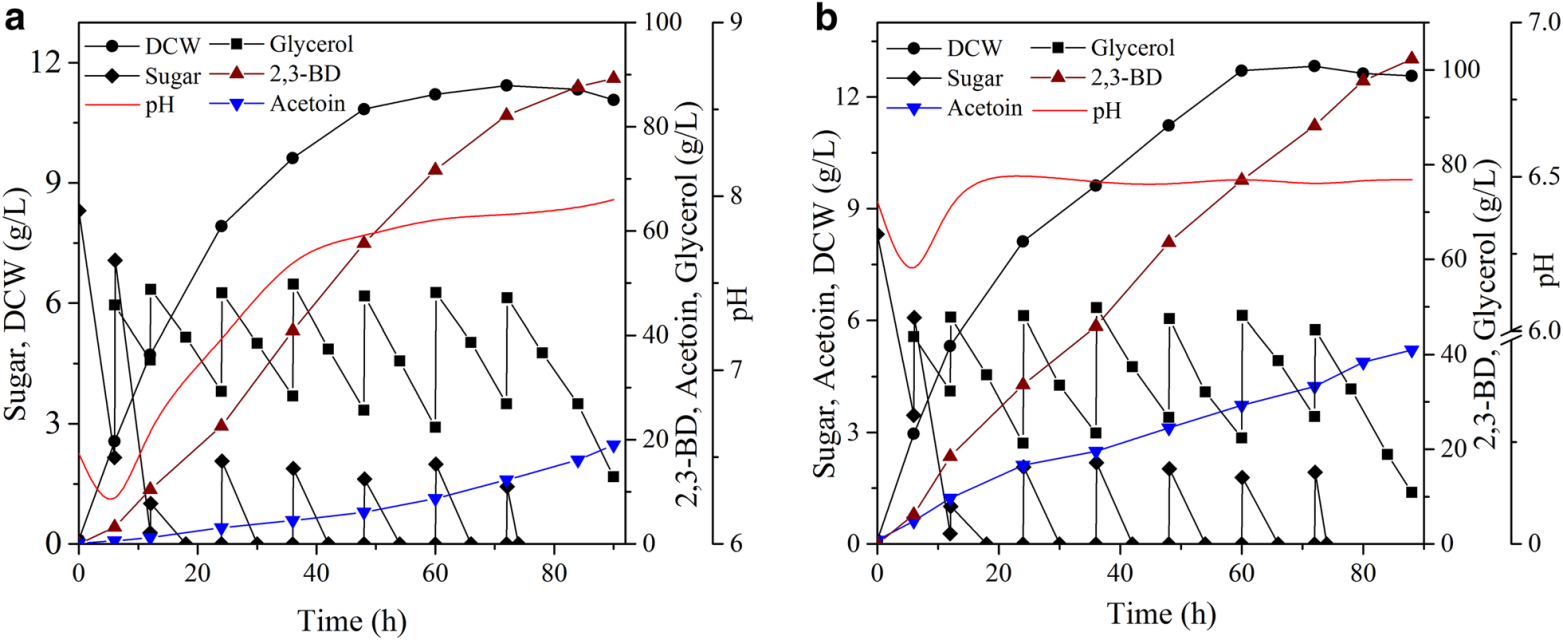
Effect of pH control strategy on 2,3-BD production by *B. amyloliquefaciens* GAR in fed-batch fermentation. **a** Without pH control; **b** At the first 16 h, the pH value was not under controlled, and after that point, pH was adjusted to 6.5.)

Firstly, fed-batch fermentation was performed under the combined feeding strategy (initial addition of beet molasses and later co-feeding with glycerol and molasses) [11]. The representative time courses of fed-batch fermentation by *B. amyloliquefaciens* GAR are presented in Fig. 4a. The 2,3-BD reached 89.5 g/L within 90 h, however, it was accompanied by undesirably large production of acetoin (~20 g/L). However, as shown in Fig. 4a, since glycerol is rapidly synthesized within 80 h of fermentation, it was also found that time profiles of 2,3-BD production could be divided into two stages. At the first 24 h, pH values were below 7.0, and 2,3-BD was quickly accumulated, while acetoin was produced very slowly. After this point, the pH value gradually rise to about 8.0, which suppressed 2,3-BD formation, while enhanced acetoin accumulation. Since 2,3-BD is produced from pyruvate in a mixed acid fermentation process, the first pH decline may be related to rapid secretion of organic acids (such as lactate and acetate). Subsequent 2,3-BD synthesis reverses the intracellular acidification and raises the pH [27].

Another major influence on 2,3-BD production is the pH [3]. Nakashimada et al. [27] reported that 2,3-BD synthesis is induced under acid supplementation, which may suggest that 2,3-BD, as a neutral metabolite, counteracts too high acidification. According to Garg and Jain [12] alkaline conditions favour formation of organic acids, with a simultaneous decrease in the 2,3-BD yield. In contrary, organic acid synthesis is reduced (over tenfold) and diol synthesis is increased (3–7-fold) under acidic conditions. Biebl et al. [8] observed that in *Klebsiella* sp., at neutral pH, it synthesizes acetic acid and ethanol, but below pH 6, 2,3-BD and ethanol are produced. However, the optimum pH for 2,3-BD production strongly depends on the microorganism and substrate used. Voloch et al. [28] found the pH range from 5 to 6 was more beneficial to 2,3-BD production by *K. oxytoca*. For *E. aerogenes*, Converti et al. [21] and Perego et al. [29] all experimentally determined a pH value of 6 as the optimum for the production of 2,3-BD. Stormer [30] found that in *K. pneumoniae* a pH above 6 causes a sharp decrease in the activity of α-acetolactate synthase (one of the key enzymes in the 2,3-BD pathway). Previously, the results also clearly showed that pH-dependent 2,3-BD production from glucose of *B. amyloliquefaciens* with the maximum production was at initial pH 6.5, and in the initial stage of fermentation, it was good for 2,3-BD synthesis without external pH control [31]. Industrial-scale fermentation requires obeying safety regulations, therefore, an urgent need for class 1 microorganisms (safe) is pronounced. The Gram-positive bacterium *B. amyloliquefaciens* has been classified as GRAS (generally regarded as safe) by the US Food and Drug Administration [32]. Therefore, increasing 2,3-BD production by *B. amyloliquefaciens* is an economically valuable goal.

**Fig. 5:**
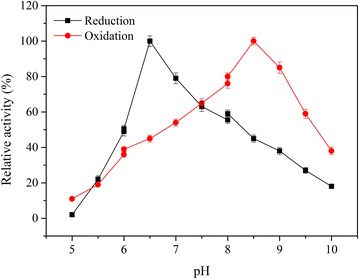
Effect of pH on acetoin reductase activity

During the production of 2,3-BD from glycerol, acetoin is the precursor of 2,3-BD. So, it is very important that maintain the suitable conditions to steadily biosynthesize 2,3-BD from acetoin. Previously, it was found that the ACR, which catalyzes the interconversion between acetoin and 2,3-butanediol [31, 33]. We further determined the effects of pH on ACR activity. As shown in Fig. 5, ACR showed very different pH preferences of pH 6.5 for reduction and pH 8.5 for oxidation. In other words, this enzyme is critical for 2,3-BD biosynthesis, exhibits the highest activity at pH 6.5, whereas at pH 7.0, about 20 % of its activity is lost, and at pH 8.0, about half of its activity is lost. This pH property of ACR from other bacterium was also reported [34–36].

Basing on the above pH preference, a two-stage control strategy was proposed to improve 2,3-BD formation (Fig. 4b). At the first stage, the pH value was not under controlled for induction of 2,3-BD formation, and then when the pH value reached about 6.5, the pH control device began to work to maintained pH value at 6.5. As expected, through the strategy, the 2,3-BD production was up to 102.3 g/L (achieved at 88 h) with corresponding productivity of 1.16 g/L h and a yield of 0.44 g/g substrate, which were 14.8, 18.4 and 15.5 % higher than that under without pH control. Thus, the two-stage pH control strategy proposed in this work is conducive to 2,3-BD formation. To our knowledge, these are the highest reported levels of 2,3-BD fermentation from biodiesel-derived glycerol.

## Conclusion

Overexpressing the NADH/NAD^+^ regeneration system effectively improved 2,3-BD production and inhibited by-products accumulation. Medorate expression of ALsR made the 2,3-BD branch gain a competitive advantage over the end products of pyruvate-deriving pathways. The carbon flux from acetoin to 2,3-BD was manipulated by using two-stage controlled pH and dissolved oxygen strategies. Finally, a high titer (102.3 g/L), yield (0.44 g/g), and productivity (1.16 g/L/h) of 2,3-BD were achieved. To our knowledge, this is the highest reported 2,3-BD production using biodiesel-derived glycerol as a substrate, and this designed *B. amyloliquefaciens* should be an excellent candidate for producing 2,3-BD on an industrial scale.

## Methods

### Strains and plasmids

Strains, plasmids and primers used in this study are listed in Table 3. The parent strain was *B. amyloliquefaciens* B10-127, which has been deposited in the China Center for Type Culture Collection (CCTCC) under collection number CCTCC M 2012349. *K. pneumoniae* ATCC 25955 was obtained from American Type Culture Collection. The recombinant derivatives of *Escherichia coli*/*B. subtilis* shuttle plasmid vector pMA5-HapII were hosted in *E.coli* JM109. This shuttle vector introduced the expression cassette for hyper-expression of *dhaD, gldA*, *acr* and *als*R into *B. amyloliquefaciens* B10-127.

**Table 1:** Effect of over-production of GDH on 2,3-BD production in *B.* *amyloliquefaciens*

Strains	C_Gly_ (g/L)	*C*_*Suc*_ (g/L)	Specific activity of GDH (U/mg)	2,3-BD (g/L)	Acetoin (g/L)	Lactate (g/L)	Acetate (g/L)	Succinate (g/L)	DCW (g/L)
B10-127	80 ± 2	10 ± 1	0.12 ± 0.01	31.3 ± 1.03	8.56 ± 0.36	2.12 ± 0.10	1.11 ± 0.06	3.65 ± 0.15	9.21 ± 0.26
DH	80 ± 2	10 ± 1	0.69 ± 0.06	34.6 ± 1.12	4.87 ± 0.21	2.46 ± 0.12	1.02 ± 0.05	3.96 ± 0.17	9.12 ± 0.21
GL	80 ± 2	10 ± 1	0.48 ± 0.04	33.3 ± 1.15	6.65 ± 0.29	2.38 ± 0.12	1.04 ± 0.06	3.81 ± 0.16	9.16 ± 0.22

*C*_*Gly*_ glycerol consumption, *C*_*Suc*_ sucrose consumption, *GDH* glycerol dehydrogenase

Batch fermentation was carried out at 37 °C in 250-mL flasks [95 g/l co-substrate; molasses to crude glycerol ratio 1.5:8 (w/w)] at 37 °C and shaking speed 180 rpm

**Table 2:** Metabolic flux distribution in *B. amyloliquefaciens* (unit: mol/mol substrate)

Strains	Flux to					
	2,3-BD	Acetoin	Lactate	Succinate	Acetate	Biomass
B10-127	0.695	0.174	0.027	0.036	0.022	0.048
GAR	0.862	0.061	0.012	0.015	0.013	0.043

**Table 3:** Strains, plasmids and primers used in this study

Bacterial strain, plasmid or primer names	Relevant characteristic or sequence	Source or enzyme site
Strains		
*B. amyloliquefaciens*		
B10-127	Host strain	Lab stock
DH	B10-127 with pMA5 -*dhaD*	This study
GL	B10-127 with pMA5-*gldA*	This study
GA	B10-127 with pMA5-*acr*-HapII-*dhaD*	This study
GAR	B10-127 with pMA5-*acr*-HapII-*dhaD*-P_bdhA_-*alsR*	This study
*K. pneumoniae* ATCC 25955	Source of *dhaD* gene	Lab stock
*E. coli* JM109		Lab stock
Plasmids		
pMA5-HapII	Expression vector (in *E. coli*, Ap^r^; in *B. amyloliquefaciens*, Kan^r^)	Lab stock
pMA5-*acr*	pMA5-HapII with *acr* (in *E. coli*, Ap^r^; in *B. amyloliquefaciens*, Kan^r^)	Lab stock
pMA5-P_bahA_-*alsR*	pMA5 containing P_bahA_-*alsR* (in *E. coli*, Ap^r^; in *B. amyloliquefaciens*, Kan^r^)	Lab stock
pMA5-HpaII-*alsR*	pMA5 containing HpaII-*alsR* (in *E. coli*, Ap^r^; in *B. amyloliquefaciens*, Kan^r^)	Lab stock
pMA5-*gldA*	pMA5-HapII with *gldA* (in *E. coli*, Ap^r^; in *B. amyloliquefaciens*, Kan^r^)	This study
pMA5-*dhaD*	pMA5-HapII with *dhaD* (in *E. coli*, Ap^r^; in *B. amyloliquefaciens*, Kan^r^)	This study
pMA5-*acr*-HapII-*dhaD*	Ap^r^, Kan^r^; pMA5-HapII with *acr* and HapII-*dhaD* (in *E. coli*, Ap^r^; in *B. amyloliquefaciens*, Kan^r^)	This study
pMA5-*acr*-HapII-*dhaD*-P_bahA_-*alsR*	Ap^r^, Kan^r^; pMA5-*acr*-HapII-*dhaD* with P_bahA_-*alsR* (in *E. coli*, Ap^r^; in *B. amyloliquefaciens*, Kan^r^)	This study
Primers		
P1	5′-CGGGATCCATGAAGCCTGAAGATATCG-3′	*Bam*HI
P2	5′-CGACGCGTCTATCCTGTCTTTTGCGC-3′	*Mlu*I
P3	5′-CGACGCGTTTTTGAGTGATCTTCTC-3′	*Mlu*I
P4	5′-CGGGATCCATGGATCGCATTATTCAATC-3′	*Bam*HI
P5	5′-CGACGCGTTTATTCCCATTCCTGCAGG-3′	*Mlu*I

Underlined nucleotides are the restriction enzyme sites

### Culture conditions

*B. amyloliquefaciens* and *E. coli* were cultured in Luria-Bertain (LB) medium. When necessary, ampicillin or kanamycin was added into the culture medium. For 2,3-BD production, *B. amyloliquefaciens* was inoculated into 10 mL LB medium added with 40 g/L glycerol and cultivated overnight with agitation (180 rpm, rotary shaker) at 37 °C. After 12 h, 2.5 mL of the seed culture (OD_600_ = 5.0–6.0) was inoculated into fermentation medium (crude glycerol (80 g/L), beet molasses (15 g/L) (Addition of beet molasses could enhance glycerol assimilation [11] ), corn steep liquor (30 g/L), soybean meal (20 g/L), ammonium citrate (5 g/L), K_2_HPO_4_ (3 g/L), MgSO_4_·7H_2_O (0.3 g/L), FeSO_4_·7H_2_O (0.05 g/L), pH6.5). Waste glycerol comprised of 88 % (w/w) glycerol, 6–9 % (w/w) water, 4–6 % (w/w) ash, 4 % (w/w) chlorides, and 0.2 % (w/w) methanol. The composition of beet molasses was 56.6 % (w/w) sucrose, 0.08 % (w/w) glucose, 23.8 % (w/w) water, 10 % (w/w) sulfated ash, 7.3 % (w/w) colloidal substances, and 2.1 % (w/w) nitrogen.

Batch fermentation was carried out at 37 °C in 250-mL flasks or a 5-L bioreactor (BIOTECH-2002, Baoxing Biological Equipment Co., Shanghai, China) containing 2.5-L initial medium (95 g/l co-substrate; molasses to crude glycerol ratio 1.5:8 (w/w)) at 37 °C and airflow rate 0.66 vvm. The fed-batch (inoculated with 4 % v/v seed culture) was cultivated in a 5-L stirring bioreactor with a working volume of 2.5 l (15 g/l molasses). Glycerol at 80 % (w/v), or a solution of 80 % glycerol, 15 % molasses, and 5 % H2O, was fed into the bioreactor to maintain the glycerol concentration between 20 and 50 g/l from 5 to 88 h. Supplementation was ceased after 88 h to minimize the glycerol residue in the final broth [11].

### Plasmids construction

The gene *dhaD* and *gldA* (encoding glycerol dehydrogenases) from *K. pneumoniae* ATCC 25955 were amplified by PCR technique using primers P1/P2 and P4/P5, respectively. The purified PCR products were separately double-digested by *Bam*H I and *Mlu* I, and then ligated to the corresponding sites of the pMA5 plasmid. The recombinant plasmid pMA5-*dhaD* and pMA5-*gldA* were generated.

The *dhaD* gene, containing the HapII promoter from the pMA5-*dhaD* plasmid, was then PCR-amplified using primers P2 and P3. The amplified HapII-*dhaD* gene was inserted into the Mlu I site of the previously constructed plasmid pMA5-*acr* [15] to create the pMA5-*acr*-HapII-*dhaD* plasmid. The P_bdhA_-*alsR* gene cut from the previously constructed plasmid pMA5-P_bdhA_-*alsR* [18] was inserted into the *Eco*R V and *Hind* III sites of the pMA5-*acr*-HapII-*dhaD* plasmid to create the pMA5-*acr*-HapII-*dhaD*-P_bdhA_-*alsR* plasmid. These constructed plasmids were isolated from *E. coli* JM109 and subsequently transformed into *B. amyloliquefaciens* according to published method [37]. Furthermore, the stability of plasmids in *B. amyloliquefaciens* was also tested as described in our previous study [15].

### Enzyme assays

The cell pellets collected by centrifugation were suspended and washed with 0.1 M potassium phosphate buffer (pH7.0) at least for three times. For determining DhaD or GldA activity [14], the cell pellets were resuspended in binding buffer (20 mM potassium phosphate, 500 mM NaCl, 20 mM imidazole, pH 7.4). For determining acetoin reductase (ACR) activity [38], the cell pellets were suspended in 0.1 M potassium phosphate buffer (pH 6.5) containing 0.1 mM β-mercaptoethanol and 2 μg/mL phenylmethylsulfonyl fluoride. For acetolactate synthase (ALS) and acetolactate decarboxylase (ALDC) enzymes, the cell pellets were washed three times with wash buffer (0.2 mM NaH_2_PO_4_, 2.2 mM Na_2_HPO_4_, and 8.5 mM NaCl; pH 7.4) and then suspended in this buffer. Cells were disrupted using a sonicator (SONICS, Newtown, CT) for 20 min with chilling. The lysed cells were centrifuged at 12,000 rpm for 25 min at 4 °C, and the supernatant was used for enzyme assays. The ALS and ALDC assays were performed according to published procedures [39].

### Analytical methods

The cell mass concentration was determined from the OD_600_ in a UV–visible spectroscopy system (UV-2000, UNICO, America). The dry cell weight (DCW) was calculated from the optical density using a calibration curve for the strain. The composition of the fermentation broth (glycerol, 2,3-BD, acetoin, acetate, lactate and succinate) was determined by high-performance liquid chromatography (HPLC) [31]. The intracellular NAD^+^ and NADH concentrations were measured by procedures described previously [40]. All assays were performed by triplicate cultures.

### Kinetic parameters calculation

The specific cell growth rate (μ_X_, h^−1^), specific substrate consumption rate (μ_S_, h^−1^) and specific 2,3-BD formation rate (μ_P_, h^−1^) were estimated from experimental or fitted data of cell growth (X, g/L), residual substrate concentration (S, g/l), and 2,3-BD production (P, g/L) by Eqs. (1)–(3), respectively. The fitted data were obtained by interposing between experimental data of cell growth, residual substrate concentration or 2,3-BD production at definite time (dt = 0.1 h) with the approximation method of cubic spline interpolation in Origin software (Version 8.0, OriginLab Corp., Northampton, MA, USA) [20].

1 $$\mu_{\text{X}} = \frac{1}{X}\frac{dX}{dt} = \frac{1}{X}\mathop {\lim }\limits_{\varDelta t \to 0} \frac{\varDelta X}{\varDelta t}$$

2 $$\mu_{\text{S}} = - \frac{1}{X}\frac{dS}{dt} = - \frac{1}{X}\mathop {\lim }\limits_{\varDelta t \to 0} \frac{\varDelta S}{\varDelta t}$$

3 $$\mu_{\text{P}} = \frac{1}{X}\frac{dP}{dt} \frac{1}{X}\mathop {\lim }\limits_{\varDelta t \to 0} \frac{\varDelta P}{\varDelta t}$$
